# Evaluation of a psychosocial education program for families with congenital adrenal hyperplasia

**DOI:** 10.1186/1687-9856-2013-S1-P119

**Published:** 2013-10-03

**Authors:** I Mitchelhill, G Betts, J King, F Murray, J Crisp, C Briggs

**Affiliations:** 1 Endocrinology, Sydney Children's Hospital, RANDWICK, NSW, Australia; 2 Sydney Nursing School, NSCCH, University of Sydney, Camperdown, NSW, Australia; 3 Sydney Nursing School, University of Sydney, Camperdown, NSW, Australia; 4 Faculty of Nursing Midwifery & Health, University of Technology, Sydney, NSW, Australia

## 

Congenital Adrenal Hyperplasia (CAH) is an inherited condition caused by an enzyme deficiency which leads to a potentially life threatening adrenal crisis. Poor compliance and any illness, injury, or major medical procedure can be life threatening. Medical interventions, counselling and timely education are essential for these families.

This study evaluated a psychosocial education program (PEP) developed to meet the needs of families with a child with CAH, with the additional opportunity of exploring the impact of CAH on the child and family.

Two hundred and two participants (parents/carers/children with CAH and siblings), from 68 families took part in the study. Participants attended a full-day workshop which included information about CAH, a practical injection session, group discussions and provision of a resource folder. Data were collected to explore the impact of CAH on the child and family using the Child Behaviour Checklist (CBCL) and the Child Health Questionnaire (CHQ). Sibling data were collected for comparison of the CAH group with siblings and population norms. Evaluation of the PEP involved baseline and follow-up (immediate, six months and 12 months post education) measures of knowledge using the CAH Knowledge Assessment Questionnaire (CAHKAQ) and a formal evaluation.

Evaluation of the PEP showed that knowledge increased immediately following the PEP, which was maintained over time. Sick day management was seen to be the major challenge for families. Fathers' and Mothers' scores for behaviour (CAH group compared to siblings) were within population norms, although Father's rated children with CAH, having lower social competence than siblings and norms. Fathers' and Mothers' were in agreement about the impact of CAH, with ratings lower than siblings and population norms for bodily pain & discomfort, general health of the child, and emotional impact on parents. The PEP achieved its major goal of increasing knowledge about CAH, and was positively evaluated by families. The workshop has now been incorporated into a DVD that is available to families and health professionals.

